# The association between levels of alcohol consumption and mental health problems and academic performance among young university students

**DOI:** 10.1371/journal.pone.0178142

**Published:** 2017-06-28

**Authors:** Chimwemwe Tembo, Sharyn Burns, Fatch Kalembo

**Affiliations:** 1 Saint. John of God Hospitaller Services, Lilongwe, Malawi; 2 School of Public Health, Curtin University, Perth, Western Australia; 3 Faculty of Health Sciences, Mzuzu University, Mzuzu, Malawi; University of Westminster, UNITED KINGDOM

## Abstract

**Purpose:**

Mental health problems and harmful alcohol consumption have been found to be high among young university students compared to the general population in Australia. This research aimed to investigate the association between levels of drinking and mental health problems and academic performance among university students aged 18 to 24 years.

**Methods:**

This study used a quantitative cross-sectional design using data that were collected in 2014 as part of the Youth Alcohol Project (YAP). Participants were randomly drawn from a cross sectional sample of 6000 undergraduate students. Included in the study were only students who were within the age of 18–24, undergraduate, and internally enrolled at the main campus. A total of 2518 undergraduate students aged 18 to 24 years who were enrolled internally at Curtin University Bentley campus were randomly recruited. Data were collected through an online survey. Students were invited to participate in the study through their student email address. The email invitations coincided with the release of semester results to increase the likelihood of students accessing their emails. A further 628 students were randomly recruited through face to face intercept survey during the campus market days. Data were collected by trained research assistants. Validated instruments were used to collected data on levels of alcohol consumption, mental health, and academic performance.

**Results:**

A considerable proportion of participants (44%) reported consuming alcohol at hazardous or harmful levels. Multiple logistic regression analysis showed that students who were consuming alcohol at hazardous levels were 1.2 times more likely to report psychological distress than those with lower levels of alcohol consumption (aOR 1.2, 95% CI: 1.1–1.5). In addition, being late for class (aOR 1.7, 95% CI:1.1–2.4), missing classes (aOR = 2.6, 95% CI: 1.9–2.6), inability to concentrate in class (aOR = 2.6, 95% CI: 1.9–3.4), and inability to complete assignments (aOR = 3.5, 95% CI 2.0–6.0) independently predicted for moderate or hazardous alcohol consumption.

**Conclusion:**

The study shows that a considerable proportion of undergraduate students at university consume alcohol at hazardous or harmful levels. In addition, high levels of alcohol consumption are associated with poor academic performance and mental health outcomes among students. The results of the study warrant multi-strategy interventions that focus on policy, organisational, educational, environmental and economic strategies that will help to reduce alcohol related harms among university students.

## Introduction

Alcohol-related harm and mental health problems are among the leading public health issues in today’s society [1, 2]. For many young Australians, alcohol plays an important role in their social life [3,4]. Twenty-one percent of young Australians aged 18–24 have been found to engage in risky drinking [5] and this behaviour puts them at greater risk of short and long-term harms associated with alcohol consumption [3]. Young university students have been found to experience greater levels of alcohol-related harm compared to their non-university attending peers [6, 7]. The university environment provides a unique social context that often promotes excessive drinking [8]. Additionally, young students may be influenced by a range of developmental, environmental, and lifestyle changes which may be associated with ‘rites of passage’ [7]. The environment, both psychological and physical, also influences alcohol consumption [9]. For example, alcohol advertising, alcohol related events at universities and pressure to belong compounded by new independence are some of the attributing factors to risky drinking behaviours [9]. The Tertiary Health Research Intervention study (THRIVE) found 90% of university students (17–24 years old) consumed alcohol in the last 12 months of the study with average volumes for a typical drinking session being 5.09 standard drinks for females and 8.68 for males [8]. In addition, 48% of students aged 17–24 years exceeded the threshold for acute alcohol-related harm at least once in the last four weeks of the survey [8].

Similarly, another Australian university found that approximately 50% of young students drank to intoxication on one or more days per week [10]. Harmful levels of alcohol consumption are associated with increased risk of both long and short-term health effects [5]. Short-term harms are found to be high among young university students as a result of episodic drinking [11]. The most common harms experienced by students are vomiting, aggression, missing classes, underachievement, financial problems, and memory loss [6]. Additionally, there is substantial research that demonstrates that harmful and hazardous alcohol consumption also contributes to arange of mental health problems and disorders including depression, anxiety, and stress or psychological distress [12,13]. Studies in Europe, the USA and Australia haveshown a high prevalence of alcohol use disorders, alcohol dependence, stress, anxiety,eating disorders and depression among university students [14–17] compared to their non-university peers [18]. The association between alcohol and mental health is bi-directional with studies suggesting that individuals who are predisposed to harmfulalcohol consumption are prone to episodes of stress, depression, and anxiety [13]. Poor mental health among university students has been associated with academic pressure and irregular sleep patterns [7] and has a negative effect on academic outcomes [19]. A study conducted in Canada and the USA found that mental health problems contribute to the seven top ten barriers to academic performance [17,20].

A range of interventions that are universal, selective, or indicated are recommended to address alcohol-related problemsand mental health [21]. For example instance,university-based early intervention programs have shown to effectively intervene with heavier users of alcohol [21]. In addition, mental health prevention, promotion, and early intervention strategies demonstrate positive outcomes when dealing with mental health problems. Such intervention should include mental health literacy (knowledge and beliefs about mental disorders that aid their recognition management and prevention) [22,23]. For example, a strategy of the Youth Alcohol Project was Mental Health First Aid (MHFA) training to increase mental health literacy among nursing students [24,25]. The intervention aimed to train participants to consider signs and symptoms of mental health problems and to enable them to provide an appropriate response to someone experiencing a mental health problem or crisis until professional help was available [25]. The study found significant improvements in knowledge scores, confidence in helping, mental health first aid intentions, stigma and social distance for the intervention compared to control group participants [26]. Literature provides evidence that the university setting offers an environment that promotes excessive drinking [9] and young university students are at increased risk of mental health related issues.

Therefore, there is a need to explore the extent of the burden of mental health problems in relation to the level of alcohol consumption and identify the predictors to hazardous drinking so that it guides interventions that decrease risk and improve psychological wellbeing of students in universities. The aim of this paper is to describe the association between levels of alcohol consumption and mental health problems among students aged between 18–24.

## Methodology

### Study design and sample size

Cross-sectional data were collected from a random sample of undergraduate students as part of the Youth Alcohol Project (YAP) [11]. The YAP aimed to promote a culture within the university that supports responsible levels of alcohol consumption. The sample included undergraduate students aged 18 to 24 years who were enrolled internally at Curtin University Bentley campus.

Participants were randomly recruited through the University Survey’s Office and via a face to face intercept survey. An online questionnaire was emailed randomly to a total of 6000 student through their university email address. Two follow-up emails were sent to maximise participation rates. A total of 1930 students completed the survey online (response rate 32.2%), and an additional 628 students were recruited via face to face intercept survey during campus market day. The total sample of students recruited that provided completed questionnaires was 2518.

### Instrumentation

The survey collected demographic, levels of alcohol consumption, mental health, and academic performance data. Demographic data were collected using a general questionnaire that included the following information age, gender, Faculty, residence, employment status, and domestic or international student status. Levels of alcohol consumption were assessed using the Alcohol Use Disorders Identification Test (AUDIT) [27, 28]. The AUDIT has been described as an accurate tool to detect alcohol dependence among university students [29, 30]. Cross-national standardisation of the AUDIT was validated in primary health care settings in six countries (Cronbach alpha 0.87) [29].

AUDIT is a 10 item questionnaire that uses an overall harm score to categorise drinking levels into four risk levels namely: 0–7 low risk, 8–15 risky or hazardous, 16–19 r harmful and 20 and high risk [27]. Consistent with other studies, this study used the 10 item AUDIT and computed to binary variables: low-risk levels of consumption < 8 or hazardous levels of alcohol consumption ≥8 [11, 31]. Mental health was assessed using the Kessler psychological distress scale (K10) [32, 33]. The K10 is a ten-item questionnaire asking questions about anxiety and depressive symptoms that an individual experienced in the past 30-days period. The scores range from 10 to 50 [34]. Consistent with other studies, psychological distress was categorised into four categories: 10–15 no to low psychological distress, 16–21 moderate distress, 22–29 high distress and 30–50 very high distress [17, 35]. A binary variable of low and moderate/high/very high categories was created from the four categories of psychological distress.

Low psychological distress category from the four psychological distress categories formed the first category of the binary variable while moderate, high and very high distress categories were combined to form a moderate/high and very high category. The K10 instrument was validated in previous studies and has been reported to have good internal reliability with Cronbach alpha score of 0.84 [36,37]. Academic problems were measured using the Academic Role Expectation and Alcohol Scale (AREAS) [38]. The four items in this scale addressed the number of times the student had been ‘late to class’, ‘missed class’, was ‘unable to concentrate’ and ‘failed to complete assignment’ as a result of their alcohol consumption for a reference period of twelve months (score range 0–16) [38]. The responses included ‘not at all’, ‘once’, ‘twice’, ‘three times’ and ‘four times or more’. The academic problem scale is a validated tool that has been used by a number of researchers to assess academic problems, and consistency was tested using coefficient alpha. The coefficient alpha was 76 [38, 39].

### Data analysis

Data were cleaned and analysed using IBM SPSS version 22.0. The general characteristics of the study participants were analysed using descriptive statistics. The dependent variable was a binary score of low risk and hazardous drinking. Chi-square was used to find the association between binary score AUDIT and independent variables of interest (psychological distress and academic performance). Using binary logistic regression, a full model including demographic variables that were significant in Chi-square analysis and the independent variables of interest were computed in one model. Odds ratios were used to determine the relative risk as well as the strength of association between the dependent and explanatory variables. All analyses were two-sided and the p-value was considered highly significant at <0.001 and moderately significant at < 0.05.

### Ethical approval

The study received ethics approval from Curtin University Human Research Ethics Committee (HR 54/2013). Written and verbal consent was sought from the study participants and documented. In addition, permission to conduct the study with students was obtained from the management of Curtin University.

## Results

### Demographic characteristics of study participants

Data were collected from 2518 undergraduate students. Half of the participants (50%, n = 1208) were aged 18–20 years, and half were aged 21-25years (n = 1214). Sixty-two percent (n = 1504) were females and 37.5%, (n = 908) were males. The majority of participants (88.7%, n = 2223) were domestic students and 11.3% (n = 283) were international. Regarding the year of study, the largest proportion of students (33.7%, n = 789) were in their first year of study. The largest proportion of students (30%, n = 696) worked 1-11hours a week and 28.6% (n = 661), worked 20 hours or more a week whilst 26% (n = 573) were not in any employment. In addition, the majority of participants lived with their parents (60.3% n = 1418) (see Table 1).

**Table 1:** Demographic characteristics of study participants.

Variable	Frequency (N)	Percentage%
Age		
18–20	1208	49.9
21–25	1214	50.1
Gender		
Male	908	37.5
Female	1504	62.1
other	9	0.4
Faculty		
Health Sciences	876	36.2
Science and Engineering	541	22.3
Humanities	537	22.2
Business school	462	19.1
Centre for Aboriginal Studies	6	0.2
International or national		
Domestic	283	88.7
international	2223	11.3
Employment hours per week		
None	573	24.8
1–5 hours	250	10.8
6–10 hours	446	19.3
11–19 hours	661	28.6
20 +	378	16.4
Years of study		
One	789	33.7
Two	698	29.8
Three	536	22.9
Four or more	321	13.7
Where participants live while at University		
Share a flat /house	590	25.1
Hall of student housing	114	4.9
Live with parents or guardians	1418	60.3
Live alone	46	2.0
Live with partner	128	5.4
Board	21	0.9
Other	33	1.4

### Descriptive statistics of alcohol consumption, mental health status, and academic performance

Table 2 describes patterns of alcohol consumption, mental health, and academic performance. Fifty-six percent (n = 1054) of the respondents reported consuming alcohol at low-risk levels while 44% (n = 761) reported consuming alcohol at hazardous and harmful levels. As regards to mental health status, 58.1% (n = 1276) reported low levels of distress, 28.9% (n = 636) reported moderate distress, and 13% reported high/ very high psychological distress. During the past 12 months, (16.3%, n = 303) of respondents reported being late at least once, (22.8%, n = 422) had missed a class, (25.2%, n = 466) reported to be unable to concentrate in class, and (6.8%, n = 127) had failed to complete an assignment on time as a result of alcohol consumption.

**Table 2:** Descriptive statistics alcohol consumption, mental health status and academic performance.

Variable	Frequency	Percentage
Alcohol consumption		
Low level	1054	55.9
hazardous Level	679	36.0
Harmful level	154	8.2
Mental health status		
Low psychological distress	1276	58.1
Moderate psychological distress	636	28.9
High psychological distress	226	10.3
Very high psychological distress	59	2.7
Been late for classes		
Not at all	1550	83.6
Ever been late	303	16.4
Missing classes		
Not at all	1431	77.2
Ever missed classes	422	22.8
Unable to concentrate in class		
Not at all	1387	74.9
Unable to concentrate	466	25.1
Failed to complete assignment		
Not at all	1726	93.1
Ever failed to complete	127	6.9

### Demographic factors associated with moderate or hazardous levels of alcohol consumption

When variables were compared age, gender, international/national, student status, residential status and hours in paid work were significantly associated with hazardous or harmful levels of alcohol consumption (p<0.05) (see Table 3). However, there was no significant association between alcohol consumption and Faculty of study.

**Table 3:** Demographic factors associated with moderate or hazardous levels of alcohol consumption in bivariate analysis.

Variable	Low Risk N %	Hazardous/harmful N %	p- value
Age			
18–20	563 (59.0)	391 (41.0)	0.007*
21–25	607 (65.1)	326 (34.9)	
Faculty			
Health sciences	429 (62.5)	257 (37.5)	0.116
Science and Engineering	245 (59.8)	165 (40.2)	
Humanities	285 (66.0)	147 (34.0)	
Curtin Business school	210 (59.2)	145 (40.8)	
Centre for aboriginal studies	1 (25.0)	3 (75)	
Gender			
Males	405(57.5)	299 (42.5)	0.003*
Females	761 (64.8)	413 (35.2)	
International/domestic			<0.001**
international	157 (88.2)	21 (11.8)	
Domestic	1013 (59.3)	696 (40.7)	
Residence while at university			<0.001**
Share a flat/house	274 (56)	209 (43.3)	
Student housing/hall of residence	46 (51.1)	44 (48.9)	
Live with parents	728 (64.1)	407 (35.9)	
Live alone	19 (59.4)	13 (40.6)	
Live with partner & children	82 (76.6)	25 (23.4)	
Board	7 (38.9)	11 (61.1)	
others	14 (63.6)	8 (36.4)	
Hours spent on work			<0.001**
None	282(69.1)	126 (30.2)	
1–5 hours	145 (70.4)	61 (29.6)	
6–10 hours	228 (62.6)	136 (37.4)	
11–19 hours	339 (59.3)	233 (40.7)	
20+	176 (52.2)	161 (47.8)	

** p <0.001;

* p <0.01

### Bivariate association between moderate or hazardous alcohol consumption and academic performance and mental health

Psychological distress was significantly associated with hazardous or harmful levels of alcohol consumption (p< 0.001). Participants who reported moderate/ high distress were more likely to have consumed alcohol at hazardous or harmful levels. (See Table 4). All four academic areas; being late for classes (p = <0.001) missing classes (p< 0.001), unable to concentrate in class (p = < 0.001) and failure to complete the assignment (p = <0.001), were significantly associated with hazardous or harmful levels of alcohol consumption.

**Table 4:** Bivariate association between moderate or hazardous alcohol consumption and academic performance and mental health.

Academic AREAS	Low Risk N (%)	Hazardous/harmful N (%)	p- value
Been late			<0.001**
Not at all	1074 (69.3)	476 (30.7)	
Ever been late	78 (25.7)	225 (74.3)	
Missing classes			<0.001**
Not at all	1029 (71.9)	402 (28.1)	
Ever missed classes	123 (29.1)	299 (70.9)	
Unable to concentrate			<0.001**
Not at all	1006 (72.5)	381 (27.5)	
Unable to concentrate	146 (31.3)	320 (68.7)	
Failed to complete assignment			<0.001**
Not at all	1129 (65.4)	597 (34.6)	
Ever failed to complete	23 (18.1)	104 (81.9)	
K-10 (mental health)			
Low psychological distress	727 (65.3)	386 (34.7)	0.002*
Moderate psychological distress	308 (57.4)	229 (42.6)	
High psychological distress	112 (59.3)	77 (40.7)	
Very high psychological distress	23 (47.9)	25 (52.1)	
K10 binary (mental health)			
Low psychological distress	727 (65.3%)	(386)34.7%	<0.001**
Moderate/high/very high psychological distress	443 (57.2)	331 (42.8)	

** p <0.001;

* p <0.01

### Multivariate association between moderate or hazardous alcohol consumption and mental health and academic performance

When all factors were considered, being late for class, missing classes, inability to concentrate in class, inability to complete the assignment and moderate or high distress independently predicted hazardous or harmful alcohol consumption. Students who were late for classes were 1.7 times more likely to consume alcohol at hazardous or harmful levels than those who did not (aOR 1.7, 95% CI: 1.1–2.4). In addition, students who missed classes were 2.6 times more likely to consume alcohol at hazardous or harmful levels than those who did not miss classes (aOR = 2.6, 95% CI: 1.9–2.6). As regards to concentration in class, students who were unable to concentrate in class were 2.6 times more likely to consume alcohol at hazardous or harmful levels than those who did not (aOR = 2.6, 95% CI: 1.9–3.4). The multivariate regression model also found that students who failed to complete assignments were 3.5 times more likely to drink alcohol at hazardous or harmful levels than those who did not (aOR = 3.5, 95% CI 2.0–6.0). In terms of the association between mental health and hazardous or harmful levels of alcohol consumption, students who reported moderate or high distress were 1.3 times more likely to consume alcohol at hazardous or harmful levels (aOR = 1.3 95% CI: 1.1–1.7) compared to those with low psychological distress (see Table 5).

**Table 5:** Multivariate association between hazardous or harmful alcohol consumption and academic performance and mental health.

Variable	Unadjusted odds ratio uOR	95% CI	P value	Adjusted odds ratio aOR	95% CI	p- value
Late for classes						
Not at all	1.0			1.0		
Been late	6.5	5.0–8.6	<0.001	1.7	1.1–2.4	0.008*
Missed classes						
Not at all	1.0			1.0		
Missed classes	6.2	4.9–8.0	<0.001	2.6	1.9–2.6	<0.001**
Unable to concentrate in class						
Not at all	1.0			1.0		
Unable to concentrate	5.8	4.6–7.3	<0.001	2.6	1.9–3.4	<0.001**
Not able to complete assignment						
Not at all	1.0			1.0		
Not completing assignment	2.7	1.8–3.9	<0.001	3.5	2.0–6.0	<0.001**
K10 Binary						
Low distress	1.0			1.0		
Moderate/high/very high distress	1.4	1.2–1.7	<0.001	1.3	1.1–1.7	0.009*

** p <0.001;

* **p <0.01**; 1.0 = Reference group.

Adjusted for all variables in the table as well as age, gender, residential status, student status (international or domestic) participation in work for 11–19 hours and participating in more than 20 hours.

## Discussion

The aim of this study was to explore the association between levels of alcohol consumption and mental health problems and academic performance among university students between the age of 18–24 years. The study findings show that 38% of the participants consumed alcohol at a hazardous level. Males were more likely to drink at a hazardous level compared to females. The findings are consistent with findings from other studies conducted in the United States [40], New Zealand [38] and Australia [8] where males have reported consuming alcohol at hazardous levels than females. Being male was a significant predictor of hazardous drinking [11]. Slightly more participants were females which is consistent with findings from other cross-sectional studies previously conducted in Australia and New Zealand universities [6, 11, 38].

The results show a significant association between the levels of alcohol consumption and mental health problems and academic performance. Students who consumed alcohol at hazardous levels were more likely to experience moderate / high psychological distress and more likely to experience academic problems. The findings are consistent with a study by Hallett and colleagues where students who consumed alcohol at hazardous levels were more likely to experience academic problems which had a negative impact on learning [39].

Furthermore, the findings are also consistent with a study by Stallman [17] that examined the psychological distress among university students. The results showed a significant difference between levels of psychological distress and student’s Grade point average (GPA). Thus, for each increase in psychological distress, students showed lower GPA. These findings, along with those of this study suggest a need for comprehensive early intervention to address mental health among university students.

A significant difference was found between the age and levels of drinking. The study found that the proportion of students who were between 18–20 years were found to drink more hazardously (41%) compared to those between the ages of 21–25 years (34.9%). Contrary to this, an earlier study at the same university found no significant difference between these same age groups and levels of consumption [11]. However, a 2012 study found young people aged 17–19 years were significantly more likely to consume alcohol at hazardous levels than those aged 20–25 years [8]. These findings are similar to the findings of this study whereby younger students aged between 18 and 20 years were more likely to drink at hazardous levels compared to the older age group. This is an important finding given the evidence that drinking at a younger age may lead to risky drinking behaviours in later years [41]. This calls for focused and comprehensive interventions that target higher risk younger drinkers.

The majority of students in this study were domestic students. There was, however, a significance difference between being an international or domestic student and level of alcohol consumption (p<0.001). Domestic students were found to consume alcohol more at hazardous levels (mean score 5.9, SD 5.7) compared to international students (mean score 3.4, SD 3.7). This may be because some international students may come from countries with different attitudes and beliefs associated with alcohol consumption [23]. However, this issue warrants further investigation [23]. When binary variables were created from Kessler10 [18], this study found 41.9% (n = 921) of participants reported moderate to high psychological distress. The proportion of psychological distress among university students is reported to be higher (41.9%) compared to the general Australian adult population (29%) [42]. However, the prevalence of university students with psychological distress is lower in this study compared to two other studies that were conducted in Australia using Kessler 10 (53%; n = 384) in 2008 [20] and in 2009 (45%; n = 1163) [43] but, higher than a 2010 study (29%; n = 6479) [17]. Despite these differences, all these studies suggest concern regarding mental health problems among university students.

The findings of this study suggest universities, should be considered as an ideal setting for implementing mental health promotion programs that would aim to increase the awareness of mental health issues and responsible alcohol consumption. In addition, interventions should be youth friendly in order to create an environment that reduces the stigma associated with mental health problems and provide easy access to mental health services. Furthermore, the findings also highlight the need to address the mental health needs of young female students who are more likely to have a higher prevalence of mental health problems associated with hazardous drinking. Hence, there is a need to design interventions that are universal to minimise stigma associated with mental health problems, selected interventions that are gender-focused, and indicated interventions that would assist students that are experiencing problems as a result of hazardous drinking. Individual oriented interventions like screening and brief motivational counseling will be necessary. Examples of such interventions have been implemented in some Australian Universities. For example “staying on Track” implemented in Queensland [17].

The limitations of this study should be considered when interpreting the results. Mental health problems in this study were limited to anxiety and depression symptoms and therefore did not encompass the full spectrum of mental health problems that students may experience. While the K10 is widely used to measure population-level mental health, especially anxiety and depression, it does not provide true diagnosis [17]. Respondents were more likely to be females compared to males and were more likely to be enrolled in the Health Science Faculty. This Faculty has more females enrolled; females have been found to be more likely to respond to university surveys than men [44]. The cross-sectional design was not the most robust design to assess the association between mental health and levels of drinking due to the inability to establish causal relationships [11]. Additionally, the results are specific to one university and this limits generalisation of the results [11]. Thus, the results may not be comparable with other international studies, because of the different social, and institutional contexts, screening instruments, and even survey methodologies that were used [18].

This paper has described a significant association between the levels of alcohol consumption and mental health problems. In addition, the results also highlight that there is an association between mental health and academic problems and that students with moderate /high psychological distress were more likely tore port not completing assignments. The findings from this study are very comparable with other studies which demonstrate that students who report moderate to high psychological distress are more likely to experience academic problems. Further research to explore the service utilisation and barriers to accessing mental health service is essential to address mental health issues and ensure young university students access available services. The relationship between alcohol outlets to excessive drinking and alcohol-related problems should also be further explored. This research has contributed to the body of knowledge related to the assessment of the prevalence of alcohol consumption in universities. While there is evidence that overall prevalence of alcohol consumption is slightly lower among young Australians [37], the proportion of hazardous drinking among university students in this study is still high. Therefore, the current results describing prevalence provide additional information supporting interventions for reducing alcohol consumption among young people. The results also justify the need to consider the university setting as an ideal environment for health promotion interventions.
